# The role of social support and social identification on challenge and threat cognitive appraisals, perceived stress, and life satisfaction in workplace employees

**DOI:** 10.1371/journal.pone.0288563

**Published:** 2023-07-12

**Authors:** Jamie C. Gillman, Martin J. Turner, Matthew J. Slater

**Affiliations:** 1 School of Health and Society, University of Salford, Manchester, United Kingdom; 2 Department of Psychology, Manchester Metropolitan University, Manchester, United Kingdom; 3 School of Health, Science and Wellbeing, Staffordshire University, Stoke-on-Trent, United Kingdom; Wilfrid Laurier University, CANADA

## Abstract

There is an emergent literature highlighting the positive role of social support and social identification in buffering against the deleterious effects of psychological stressors. Yet, we have limited understanding of how exactly these social factors fit within contemporary stress and coping theory. To advance and gain a greater understanding of these social factors, we explore the associations of social support and social identification on individuals’ challenge and threat cognitive appraisals and how this then relates to perceived stress, life satisfaction, turnover intentions, and job performance. A total of 412 workplace employees from private and public sector occupations completed state measures around a recent most stressful experience at work. Results revealed atemporal associations between cognitive resource appraisals with both social support and social identification. Specifically, greater identification with colleagues and lower threat were related to less perceived stress, while having greater social identification (with colleagues and organisation), social support, and lower threat, were related to greater life satisfaction. Greater perceived stress, and lower social identification and life satisfaction, were also related to greater turnover intentions. While greater identification with the organisation and life satisfaction, along with lower perceived stress were related to greater job performance. Taken together, this research provides evidence that social support and social identification play a positive role when trying to promote more adaptive responses to stressful situations.

## Introduction

Stress is ubiquitous across all occupational domains and typically individuals who experience greater levels of stressors in the workplace are more likely to be unhealthy, poorly motivated and less productive [1]. Workplace stress is defined by the World Health Organization as “the response people may have when presented with work demands and pressures that are not matched to their knowledge and abilities and which challenge their ability to cope” [1 p. 3]. Stress can have maladaptive consequences to health and well-being. For example, work stress has consistently been associated with both poorer psychological and physical health, with distinct links to anxiety and depression, and physical side-effects such as migraines, injury, exhaustion, and disturbed sleep [2–4]. The most recent Health and Safety Executive (HSE) report in Great Britain recorded an estimated 17 million working days were lost due to work-related stress, depression or anxiety, and accounted for over half of all work-related ill health cases in 2021/22 [5]. The economic costs to the British society as a result of work-related stress is considerable, with it being estimated to be around £5.2 billion every year [6]. The causes of workplace stressors can vary and be unique to a work organisation or industry, but examples include unreasonable performance demands, lack of autonomy and control over work, unclear roles, responsibility, and job insecurity [1, 7]. How an individual responds and copes with workplace stressors can be variable and not always seen as debilitating, as some work-related stress may actually increase motivation and performance [8, 9].

Dominant in the stress and coping literature are transactional models of stress, in which stress occurs as an interaction between the individual and the environment, influenced by both primary (i.e., identifying potential danger) and secondary (i.e., coping) appraisals [10, 11]. Drawing from the appraisal theory, researchers have been interested in the human stress response in a variety of domains and within specific motivated performance situations (e.g., interviews, sporting performances, exams). One established theory that provides further detail in the area of stress and coping is the biopsychosocial model of challenge and threat (BPSM [12]). In the BPSM, it is proposed that in motivated situations (e.g., interview performance), individuals make two distinct cognitive appraisals: demand and resource appraisals. Demand appraisals refer to the perception of danger, uncertainties, and required effort of the situation, while resource appraisals refer to the perceived resources and abilities to deal with the situation (e.g., skills, knowledge, abilities, and dispositional factors). Accordingly, these cognitive appraisals determine whether an individual evaluates a situation as a challenge or threat. Challenge (adaptive) occurs when the perceived resources meet or exceed the perceived demands of the situation. In contrast, threat (maladaptive) occurs when the perceived resources do not meet the perceived demands.

Since the formulation of the BPSM, several scholars have adopted challenge and threat as a framework to better understand the human stress response. For example, the Theory of Challenge and Threat States in Athletes (TCTSA [13]) was developed to understand athletes’ responses to a competition and the impact it has on performance outcomes through their cognitions, emotions, and physiological responses. Extending the BPSM by introducing three interrelated resource appraisals (i.e., self-efficacy, perceptions of control, and achievement goals), the TCTSA also outlined emotional states relating to challenge and threat by suggesting that positive emotions are typically associated with challenge, and negative emotions typically with a threat state [13]. A growing body of research has adopted the BPSM and TCTSA frameworks to explore challenge and threat in an array of different contexts such as coping with stereotype threat [14], classroom presentations [15], exams [16], and laparoscopic surgery [17]. Of particular interest to researchers are performance outcomes, and studies have shown that a challenge state is related to superior performance compared to a threat when approaching a motivated performance situation [18, 19]. However, challenge and threat theories such as the BPSM and the TCTSA have largely focused on egocentric appraisals of situational demands and resources, excluding socially derived perceptions. More recently, the TCTSA has been revised (TCTSA-R [20]) which re-evaluates the resources, specifically to consider the inclusion of social support. However, there is currently little empirical evidence examining this notion.

It has been noted that the literature on stress and coping is dominated by individualistic approaches that have neglected the social aspects [21]. Human beings are social mammals and have a need to belong [22], as well as a need to be competent and autonomous [23]. Thus, it is necessary that social factors are considered when examining psychological stress. More recently, researchers’ have recognised the importance of social factors in the transactional stress process. A key social factor that can influence how a person manages stress is an individual’s perceptions of social support, which has reputed benefits to physical and psychological health [24].

Social support can be defined as “support accessible to an individual through social ties to other individuals, groups, and the larger community” [25 p. 109]. House [26] outlined social support as the functional content of relationships that can be determined by four broad categories of supportive behaviours or acts. These include emotional support (i.e., empathy), instrumental or tangible support, (i.e., provision of material aid) and appraisal support (i.e., provision of information that is useful for self-evaluation). There have been several variants of the type of social support although Cutrona and Russell [27] outlined the four which has received most agreement as being emotional, esteem, informational, and tangible support. Social support can also be regarded as verbal or non-verbal (i.e., nodding, smiling, eye contact) and separated into perceived and received categories. Perceived support refers to a person’s potential access to supportive resources and is independent of the actual reception of support [28], whereas, received support refers to actual support that a person receives [29, 30].

Social support has been found to improve physical and psychological health [24, 31], alongside acting as a buffer to stress [32]. Two key models underpin these outcomes: (1) the direct-effects (also called main effects) hypothesis which proposes that social support is beneficial all the time regardless of whether the supported person is experiencing stress or not; and (2) the buffering-effect hypothesis, which proposes social support having more of an influence on the factors related to a stressful situation [28]. Researchers have shown that individuals with low levels of social support have higher mortality rates, in particular from cardiovascular disease [33], while high levels of social support have been linked with lower mortality rates from cancer [34], HIV [35], increased psychological well-being in the workplace [26], and greater life satisfaction [36]. Nevertheless, these results have been seen to differ for both perceived and received support. For instance, perceived support is consistently associated with positive health outcomes [24, 37, 38], while, received support has often shown inconsistent effects on health, and even negative outcomes have been found [24].

Social support is also thought to intervene in the stress process by affecting secondary appraisal (i.e., the person’s ability to cope with a stressor). For example, adequate support may lessen the impact the stress appraisal has, by providing a solution to the problem, or, by reducing the perceived importance of it [32]. Social support can also act as a useful resource and is apparent in various forms such as emotional support (i.e., empathy and acceptance), instrumental/tangible support, (i.e., provision of material aid) or appraisal/informational support (i.e., provision of information that leads to alternative assessments of the stressor itself or one’s ability to cope with it) [26, 32]. A study among police officers found that that the social support between co-workers significantly buffered the relationship between work-related events and distress [39]. Social support then is likely to increase individuals’ perceptions of being able to deal successfully with stressors as they can draw upon and utilise collective actions [40]. For example, talking to a co-worker about a stressful situation can act as a problem-focused coping strategy drawing upon the various forms of support. In another study, Dixon et al. [41] explored the relationships between challenge and threat cognitive appraisals and coaching behaviors in football coaches and found that coaches with a tendency to appraise a stressor as a challenge are more likely to offer social support to their athletes. This suggests a reciprocal relationship between challenge and threat appraisals and social support, meaning those who display a challenged state perhaps have more capacity to offer support to others because they can cope with the demands of the situation.

Researchers have also suggested that social support may be a valuable resource to encourage challenge states particularly when underpinned by high social identification [42]. Social identification can be defined as the extent to which an individual feels they belong to a group (e.g., an organisation, a work team, leisure group) [43, 44]. Social Identity Theory (SIT [44]) suggests that in social contexts people can define themselves as individuals (i.e., personal identity; ‘I’ and ‘me’) and as group members (i.e., social identity ‘we’ and ‘us’). In other words, personal identity reflects an individual’s perception of themselves to be distinct and different from other people in an environment, while social identity refers to “that part of an individual’s self-concept which derives from his membership of a social group (or groups), together with the value and emotional significance attached to this” [45 p. 63]. Alongside SIT, within Self-Categorisation Theory (SCT [45]) it is asserted that an individual’s sense of self is informed by their group membership and therefore appraisal of stressors will be affected by other members of their ingroup. In other words, how an individual first appraises and consequently copes with a stressor can be influenced by shared group membership. More recently, the sociopsychobio model [46] provides a framework to encapsulate the interplay of social, psychological, and biological factors related to health and places social identification and social support as central tenants in the stress process. As such, offering a useful framework for the current study to examine.

Scholars have found that greater levels of identification with an organization is positively related to a number of work-related outcomes such as job performance, motivation, turnover intentions, and absenteeism [43, 47, 48]. For example, social identification in the workplace can increase an individual’s sense of purpose, belonging and collective self-efficacy thus eliciting health-promoting effects [49]. However, some research has shown social identification to be detrimental to health due to associations with working long hours being negatively related with employee well-being [50]. Although, a meta-analysis conducted by Steffens et al. [51] found that social identification in organisational contexts is generally positively related to individuals’ health (r = .21). For instance, individuals who identify strongly with a certain group (e.g., their department at work) have greater overall health and well-being [49, 51, 52] and are also more likely to experience social support from other members of that group [53, 54].

Not only has social identification been seen to increase the prevalence of social support, but it has also been shown to increase the effectiveness of the support received. To illustrate, a shared social identity provides a foundation for individuals to interpret support in ways that are more beneficial and helpful to the recipient [55, 56]. For example, Frisch et al. [57] found that emotional social support buffered neuroendocrine stress reactions only if a shared social identity was established between the provider and receiver. In an organizational context, social identification can be seen as a key variable in helping individuals perceive greater support that helps them cope with stress and reduce turnover intentions [58]. That being said, past research evidence has shown that emotional social support is not always effective and sometimes has no impact on buffering against stressful situations [59, 60], or can be detrimental, leading to heightened stress reactions [61, 62]. It could be the case that received support may in fact lower self-esteem, and/or draw more attention to the problem [63]. These opposite effects are sometimes referred to as “reversed buffering effect”, and research around stressful work events have shown that social support was actually related to greater distress within the workplace [64, 65]. Thus, a shared social identity could be useful to interpret support in a more beneficial way and prevent individuals from making such implicit criticism (e.g., feelings of inequality, threat to self-esteem) [55].

### The present research

Currently, few studies have examined the associations between social support and social identification and made direct links to challenge and threat states [41, 66–68]. For example, Slater et al. [66] found that relational identification with a leader increased resource appraisals and influenced cardiovascular reactivity in line with challenge and threat theory. In a more recent study, Miller et al. [68] operationalised social support as a resource appraisal across two studies with an athletic sample. The researchers found that relational identification and group identification mediated the positive relationship between identity leadership and self-efficacy, control, approach goals and social support. However, these studies were in the context of leadership identity, so the generalisability to other domains is unknown. Challenge and threat theory offers a contemporary approach to understanding the human stress response by acknowledging both adaptive (challenge) and maladaptive (threat) responses to stressful situations. While considered comprehensive, the theory has lacked the inclusion of social factors. The BPSM had been revised to include the availability of support as an antecedent of challenge and threat [69], yet the exact mechanisms are unclear and warrants further examination [70]. Equally, the TCTSA-R [20] puts forth social support as a resource appraisal, however the evidence examining this is scant. Given that social support helps buffer against the deleterious effects of stress, especially when underpinned by social identification, it may be possible to elicit greater challenge through the reduction of perceived demands and offering a useful resource in the face of a stressful situation. Specifically, social support has been associated with an increase in psychological well-being in the workplace [26]. While high levels of work stress are associated with lower life satisfaction [71], and a number of other work-related outcomes including intentions to quit (turnover [72]), absenteeism and presenteeism (job performance [73]). Thus, gaining a better understanding of the stress response and the role of social factors is of high health, societal and economic significance.

The aim of the current study was to examine the role of social support and social identification in individuals’ challenge and threat cognitive appraisals, and the effect that this has on perceived stress and life satisfaction in workplace employees. The study aims to contribute to the literature by empirically testing the postulations put forth in contemporary stress theory (i.e., TCTSA-R) and the framework proposed in the sociopsychobio model of health [46] to examine how the social factors can influence stress within the workplace. Based on past research, we hypothesised that there would be positive relationship between social support and social identification (H1), and that greater social support and social identification would be related to greater challenge, and lower threat (H2), which in turn would be related to less stress (H3), greater life satisfaction (H4), less turnover intentions (H5), and lower absenteeism (H6), along with greater job performance (H7).

## Method

### Participants

We recruited 412 participants (female = 264, male = 148) participants (*M*age = 36.36 years, *SD*age = 11.19 years) to complete an online questionnaire on one occasion. Through purposeful sampling, participants consisted of workplace employees from a range of private and public sector occupations, to capture an array of professions within the occupational context (e.g., health, education, social work, government, services, domestic services). Participants consisted of service workers (i.e., fire & rescue, the police service, NHS, & social services; N = 179), private sector workplace employees (N = 138), and those who work in education (N = 95). A breakdown of participants job titles can be found in the S1 Table. Participants were recruited through the distribution of an online survey via social media (i.e., Twitter and Facebook), and Prolific’s participant pool. Prolific is a data collection tool which allows the distribution of questionnaires to those who meet the inclusion criteria and has been considered a valuable recruitment platform for researchers [74]. Overall, there were 549 responses to the questionnaire. Following screening for the inclusion criteria (i.e., over the age of 18, employed in the UK, written informed consent provided) and data quality (i.e., incomplete measures, unrealistic completion time compared to the mean, straight-line responses), 137 respondents were removed from the dataset. This resulted in 412 eligible participants. Of these 412 participants, 152 (36.9%) were recruited via Prolific. With a power of .80 and an alpha of .05, a target sample of 395 was deemed sufficient to detect a small effect (f^2^ = .02) according to an apriori calculation using G*Power for multiple regression analysis.

### Measures

#### Appraisal of life events scale (ALE scale)

The appraisal of life events scale (ALE-scale [75]) was used and consists of 16 adjectives in which participants were asked to rate in relation to their perceptions of their most stressful experience at work in the last three months (participants also described the event in qualitative form) on a 6-point Likert scale from 0 (*not at all*) to 5 (*very much so*). Challenge and threat is determined by taking the mean scores from two subscales. Cronbach’s alpha for the ALE-scale in the current sample was α = .66 for challenge, and α = .66 for threat.

#### Social identification

The Single-Item Social Identification (SISI [76]) measure was used to assess individual’s identification to their: (1) organisation; and (2) colleagues. The two items asked individuals to rate how far they agree with the following statement in relation to their group: “I identify with my (organisation/workplace colleagues)” on a seven-point Likert-scale ranging from 1 (*strongly disagree*) to 7 (*strongly agree*). This measure has proven to capture social identification in one item and has shown high reliability and validity in past research [76].

#### Social support

Social support was measured using the Multidimensional Scale of Perceived Social Support (MSPSS [77]). This contained three subscales of different sources of support: family, friends, and significant other. Participants were asked to rate how they felt in relation to the stressful work event across twelve statements on a 7-point Likert-scale ranging from 1 (*very strongly disagree*) to 7 (*very strongly agree*). A total social support score was created by calculating an average score for all twelve items. The MSPSS is one of the most widely used measures of perceived social support and has adequate internal consistency reliability [78]. Cronbach’s alpha for the total social support score in the current sample was α = .93 demonstrating excellent internal consistency.

#### Life satisfaction

Life satisfaction was measured using six items from the Multidimensional Life Satisfaction Scale’ (BMLSS [79]) which was developed from the Brief Multidimensional Students’ Life Satisfaction Scale (BMSLSS [80]). This contained six items assessing satisfaction with self, family, friends, living environment, school, and global life satisfaction. Although the BMSLSS was originally intended for students under the age of 18, the measure has been used in several contexts to assess outcomes in adolescents and adults [81]. One question was adapted to fit in line with the sample for the current study, as this was the only question that was in reference to being a student. Therefore, this was replaced with “workplace”, as also seen within the BMLSS. A total life satisfaction score was created by averaging the scores across the six items. Cronbach’s alpha for the total life satisfaction score from the current sample was α = .80, demonstrating good internal consistency.

#### Perceived stress

Stress was measured using the Perceived Stress Scale (PSS [82]). The ten-item measure assessed individual’s feelings and thoughts during the most stressful event identified in the last three months. Items are measured using a 5-point Likert scale 0 (*never*) to 4 (*very often*). This is a widely used psychological instrument of stress and has been well validated in a range of populations [83]. Cronbach’s alpha for the PSS in the current sample was α = .67.

#### Turnover intentions

Turnover intention was measured using 3 items developed by Colarelli [84]. A sample item is “I frequently think of quitting my job.” Responses were anchored on a five-point scale ranging from 1 (*strongly disagree*) to 5 (*strongly agree*). Cronbach’s alpha for the 3-item turnover intention measure was α = .68.

#### Absenteeism and job performance

Absenteeism and job performance items were taken from The World Health Organization’s (WHO) Heath and Work Performance Questionnaire (HPQ [85]). For absenteeism, participants estimated how many hours they worked over a four-week period. Specifically, participants were asked to indicate how many hours their employer expects them to work in a typical 7-day week, and then how many hours they actually worked in the past 28-days. The hours they are expected to work in 7-days are multiplied by four, and then the actual days they worked in the past 28-days are subtracted from that score to form the absolute absenteeism score. Thus, absenteeism is scored in terms of hours lost per month where higher scores indicate a greater absenteeism. For job performance, one item was taken from the HPQ [85]. The item asked participants “how would you rate your overall job performance on the days you worked during the past 4 weeks (28 days)?” on a scale from 0 (worst performance) to 10 (top performance). The HPQ has excellent validity and reliability and has been used in an array of workplace settings [86].

### Procedure

Ethical approval was obtained from Staffordshire University’s research ethics committee prior to data collection. An online survey was created using Qualtrics allowing the authors to distribute the measures to participants via an anonymized system. Snowballing sampling was use by posting survey links on social media (i.e., Twitter and Facebook) to allow for re-sharing of the study. In addition, respondents were collected through Prolific’s participant pool as this allowed to target specific populations (i.e., workplace employees). Participants were provided with information regarding the study and were presented with digitised informed consent prior to taking part. The online survey was conducted between January 2017 to August 2018 and took approximately ten minutes to complete.

### Analytic strategy

Data were first examined for missing values, and little’s MCAR test revealed that across each variable between .2% and 3.1% data were missing at random, χ2 = 341.39, df = 314, *p* = .138. Expectation maximisation (EM) method were used to estimate the missing values [87] to provide a complete data set for the main analyses. Data were also examined for outliers and normality to ensure data met the assumptions for parametric testing. Significant outliers with *z* scores greater than two were windsorized [88, 89], which involved replacing extreme values to reduce the influence of outliers on the subsequent analysis. Overall, 3.21% of the data were winsorized.

Data analyses were completed in two phases. First, to test H1 and H2, Pearson correlations were carried out between social support and social identification (H1), and then with challenge and threat (H2). Second, a series of hierarchical multiple regression analyses were performed to test H3 to H7. In each regression analysis, age and sex were entered at step 1, challenge and threat were entered at step 2, and social identity and social support were entered at step 3, predicting outcome variables perceived stress (H3), and life satisfaction (H4). Third, in a further two regression analyses, perceived stress and life satisfaction were entered into step 4, predicting outcomes of turnover intentions (H5), absenteeism (H6), and performance (H7). All analyses were carried out using IBM SPSS Statistics (Version 27).

## Results

Table 1 contains descriptive statistics and bivariate correlations coefficients between all study variables. No correlation coefficient exceeded .80 indicating that multicollinearity was not an issue in further analysis. In support of H1, a small yet significant positive correlation was found between social identification and social support (organisation: *r* = .10, *p* = .04, colleagues: *r* = .22, *p* < .01). Partial support was found for H2, in that there was a small yet significant negative correlation between social identification with colleagues and threat (*r* = —.10, *p =* .04). However, in contrast to H2, a small significant positive correlation was also found between social support and threat (*r* = .11, *p =* .02). A positive relationship between social support and social identification on challenge were revealed, but these were small and non-significant. No other significant relationships were found.

**Table 1:** Means, standard deviations, and bivariate correlations for all variables.

N = 412	M	SD	Scales (Cronbach’s alpha)	1	2	3	4	5	6	7	8	9	10	11	12
1. Age	36.36	11.19		-	.06	-.05	-.05	.06	.12*	-.08	-.09	-.08	-.09	.06	.11*
2. Sex	0.64	0.48			-	-.11*	.00	-.05	.06	.13**	.17**	-.08	.03	.01	.07
3. Challenge	16.48	6.79	0–5 (.66)			-	.21**	.06	.04	.05	.04	.00	-.02	-.14**	-.05
4. Threat	17.63	7.16	0–5 (.66)				-	-.06	-.10*	.11*	.30**	-.10*	.12*	-.05	-.14**
5. SI Organization	5.17	1.26	1–7					-	.48**	.10*	-.10*	.34**	-.44**	.04	.21**
6. SI Colleagues	5.63	1.21	1–7						-	.22**	-.20**	.35**	-.42**	.11*	.21**
7. Social Support	4.98	1.21	1–7 (.93)							-	.00	.37**	-.13**	.02	.01*
8. Perceived stress	21.43	5.07	0–5 (.67)								-	-.38**	.27**	-.06	-.21**
9. Life satisfaction	5.30	0.78	1–7 (.80)									-	-.36**	.10*	.27**
10. Turnover intentions	2.06	0.90	1–5 (.68)										-	-.09	-.17**
11. Absenteeism	0.23	34.53	hours											-	-.05
12. Performance	7.75	1.31	0–10												-

Note:

* *p* < .05,

** *p* < .01;

SI = Social Identification. Males were coded 0 and females were coded 1

### Predicting stress

As shown in Table 2, the hierarchical multiple regression for perceived stress revealed that all steps were significant in the model. When all variables were included in step three of the regression (R^2^ = .142, *F*(7, 410) = 10.657, *p* < .001), standardised coefficients revealed only sex (*β* = .19, *p* < .001), threat (*β* = .28, *p* < .001), and social identification with colleagues (*β* = -.17, *p* = .002) were significant predictors of perceived stress such that, females and having greater threat, and lower identification with colleagues were related to greater perceived stress.

**Table 2:** Hierarchical regression analyses for challenge and threat, social identity and social support, predicting perceived stress and life satisfaction.

	Step 1				Step 2				Step 3			
Perceived stress												
Variable	b	SE	ß	95% CIs	b	SE	ß	95% CIs	b	SE	ß	95% CIs
Age	-.046	.022	-.102	-.089, -.003*	-.039	.021	-.087	-.081, .002	-.032	.021	-.071	-.074, .065
Sex	1.902	.513	.180	.893, 2.911**	1.887	.494	.178	.916, 2.857**	2.041	.494	.193	1.070, 3.012**
Challenge					-.006	.036	-.007	-.076, .065	.003	.035	.004	-.066, .073
Threat					.210	.034	.296	.144, .276**	.200	.034	.282	.134, .266**
SI Organisation									.049	.212	.012	-.368, .465
SI Colleagues									-.708	.227	-.168	-1.155, -.261*
Social support									-.134	.201	-.032	-.528, .260
R^2^	.036** (ΔR^2^ = .041**)				.119**(ΔR^2^ = .087**)				.142**(ΔR^2^ = .029*)			
F	8.618**				14.798**				10.657**			
Life satisfaction												
Variable	b	SE	ß	95% CIs	b	SE	ß	95% CIs	b	SE	ß	95% CIs
Age	-.005	.003	-.073	-.012, .002	-.005	.003	-.078	-.012, .001	-.006	.003	-.084	-.012, .000
Sex	-.122	.080	-.075	-.280, .036	-.120	.080	-.073	-.278, .039	-.194	.070	-.119	-.332, -.055*
Challenge					.001	.006	.010	-.010, .013	-.003	.005	-.030	-.013, .006
Threat					-.012	.005	-.108	-.023, -.001*	-.011	.005	-.105	-.021, -.002*
SI Organisation									.132	.030	.212	.073, .192**
SI Colleagues									.115	.032	.177	.051, .179**
Social support									.212	.029	.329	.156, .268**
R^2^	.007 (ΔR^2^ = .012)				.013*(ΔR^2^ = .011)				.267**(ΔR^2^ = .257**)			
F	2.379				2.365				22.379**			

Note:

* *p* < .05,

** *p* < .01;

Males were coded 0, and females were coded; SI = Social identification

### Predicting life satisfaction

For life satisfaction, the hierarchical multiple regression revealed that sex and age at step one, and challenge and threat at step two did not explain a significant proportion of variance in life satisfaction. Adding social identity and social support at step three did explain a significant proportion of variance in life satisfaction (Table 2). When all variables were included in step three of the regression (R^2^ = .267, *F*(7, 410) = 22.379, *p* < .001), standardised coefficients revealed sex (*β* = -.12, *p* = .006), threat (*β* = -.11, *p* = .017), social identification with organisation (*β* = .21, *p* < .001), social identification with colleagues (*β* = .18, *p* < .001), and social support (*β* = .33, *p* < .001) were significant predictors of life satisfaction. That is, males and having greater social identification, social support, and lower threat, were related to greater life satisfaction.

### Predicting turnover intention

As shown in Table 3, the hierarchical multiple regression for turnover intention revealed that sex and age at step one did not contribute significantly to the regression model, but all the other steps were significant. When all variables were included in step four of the regression (R^2^ = .284, *F*(9, 410) = 19.052, *p* < .001), standardised coefficients revealed social identification with organisation (*β* = -.27, *p* < .001), social identification with colleagues (*β* = -.20, *p* < .001), perceived stress (*β* = .14, *p* = .005), and life satisfaction (*β* = -.15, *p* = .006), were significant predictors of turnover intention. That is, greater perceived stress, and lower social identification and life satisfaction, were related to greater turnover intentions.

**Table 3:** Hierarchical regression analyses for challenge and threat, social identity, social support, perceived stress and life satisfaction predicting turnover intentions, absenteeism and job performance.

	Step 1				Step 2				Step 3				Step 4			
Turnover intentions																
Variable	b	SE	ß	95% CIs	b	SE	ß	95% CIs	b	SE	ß	95% CIs	b	SE	ß	95% CIs
Age	-.008	.004	-.094	-.015, .000	-.007	.004	-.090	-.015, .001	-.004	.004	-.049	-.011, .003	-.004	.003	-.051	-.011, .003
Sex	.070	.093	.037	-.112, .252	.060	.093	.032	-.122, .242	.077	.082	.041	-.085, .239	-.006	.082	-.003	-.167, .156
Challenge					-.006	.007	-.047	-.019, .007	-.001	.006	-.007	-.013, .011	-.002	.006	-.012	-.013, .010
Threat					.016	.006	.124	.003, .028*	.010	.006	.080	-.001, .021	.003	.006	.026	-.008, .014
SI Organisation									-.212	.035	-.294	-.281, -.142**	-.191	.035	-.265	-.260, -.121**
SI Colleagues									-.189	.038	-.252	-.264, -.115**	-.152	.038	-.203	-.227, -.078**
Social support									-.049	.033	-.066	-.115, .017	-.010	.035	-.013	-.078, .059
Perceived Stress													.024	.009	.138	.007, .041*
Life satisfaction													-.168	.061	-.146	-.288, -.049*
R^2^	.005 (ΔR^2^ = .010)				.015*(ΔR^2^ = .015*)				.244**(ΔR^2^ = .232**)				.284**(ΔR^2^ = .042**)			
F	2.024				2.598*				19.931**				19.052**			
Absenteeism																
Variable	b	SE	ß	95% CIs	b	SE	ß	95% CIs	b	SE	ß	95% CIs	b	SE	ß	95% CIs
Age	.175	.152	.057	-.124, .475	.154	.151	.050	-.143, .452	.123	.153	.040	-.178, .423	.145	.155	.047	-.159, .449
Sex	-.109	3.546	-.002	-7.079, 6.861	-1.128	3.539	-.016	-8.085, 5.828	-1.778	3.580	-.025	-8.816, 5.259	-1.082	3.662	-.015	-8.282, 6.118
Challenge					-.690	.255	-.136	-1.193, -.188*	-.727	.256	-.143	-1.229, -.224*	-.714	.256	-.141	-1.217, -.211*
Threat					-.111	.241	-.023	-.585, .362	-.064	.244	-.013	-.544, .416	-.024	.254	-.005	-.524, .476
SI Organisation									-.385	1.534	-.014	-3.401, 2.631	-.882	1.577	-.032	-3.982, 2.218
SI Colleagues									3.199	1.647	.112	-.038, 6.436	2.778	1.680	.097	-.525, 6.080
Social support									.409	1.453	.014	-2.447, 3.266	-.383	1.556	-.014	-3.443, 2.676
Perceived Stress													.015	.386	.002	-.744, .773
Life satisfaction													3.749	2.711	.085	-1.581, 9.079
R^2^	-.002 (ΔR^2^ = .003)				.014*(ΔR^2^ = .020*)				.018*(ΔR^2^ = .012)				.019(ΔR^2^ = .005)			
F	.663				2.433*				2.101*				1.873			
Job performance																
Variable	b	SE	ß	95% CIs	b	SE	ß	95% CIs	b	SE	ß	95% CIs	b	SE	ß	95% CIs
Age	.012	.006	.101	.000, .023*	.011	.006	.094	.000, .022	.009	.006	.080	-.002, .020	.010	.006	.086	-.001, .021
Sex	.177	.134	.065	-.087, .440	.174	.134	.064	-.089, .438	.150	.132	.055	-.110, .410	.262	.133	.096	.001, .522*
Challenge					-.002	.010	-.011	-.021, .017	-.006	.009	-.031	-.025, .013	-.005	.009	-.025	-.023, .013
Threat					-.024	.009	-.131	-.042, -.006*	-.021	.009	-.116	-.039, -.003*	-.012	.009	-.067	-.030, .006
SI Organisation									.154	.057	.148	.043, .265*	.118	.057	.113	.006, .230*
SI Colleagues									.111	.061	.102	-.008, .231	.059	.061	.054	-.061, .178
Social support									.082	.054	.076	-.024, .187	.018	.056	.017	-.092, .129
Perceived Stress													-.028	.014	-.109	-.056, -.001*
Life satisfaction													.282	.098	.169	.089, .475*
R^2^	.010* (ΔR^2^ = .015*)				.023*(ΔR^2^ = .018*)				.075** (ΔR^2^ = .058**)				.112**(ΔR^2^ = .041**)			
F	3.144*				3.451*				5.721**				6.720**			

Note:

* *p* < .05,

** *p* < .01;

Males were coded 0 and females were coded 1; SI = Social identification

### Predicting absenteeism and job performance

For absenteeism, the hierarchical multiple regression revealed that only challenge and threat at step two, and social identity and social support at step three contributed significantly to the regression model. Step four did not contribute significantly to the model (R^2^ = .019, *F*(9, 410) = 1.873, *p* = .054) (Table 3).

As shown in Table 3, the hierarchical multiple regression for job performance revealed that all steps were significant in the model. When all variables were included in step four of the regression (R^2^ = .112, *F*(9, 410) = 6.720, *p* < .001), standardised coefficients revealed sex (*β* = .10, *p* = .049), social identification with organisation (*β* = .11, *p* = .039), perceived stress (*β* = -.11, *p* = .045), and life satisfaction (*β* = .17, *p* = .004), were significant predictors of job performance. That is, females, with greater identification with the organisation and life satisfaction, along with lower perceived stress were related to greater job performance.

## Discussion

The results showed, as hypothesised (H1) and in support of existing research, that there was a positive relationship between social identification and social support [56, 90]. These findings suggest that individuals who have a strong connection with a particular group (e.g., their work organisation) are also more likely to perceive social support from other members of that group [53, 90]. In this sense, the exchange of social support is always dependant on the relationship between the provider and recipient [56]. Thus, a shared identity is more likely perceived as originally intended rather than misconstrued as something else [55]. It should also be noted that this finding was found when participants were responding in relation to both identification with their organisation and identification with their colleagues.

We found some evidence for H2, in that a negative relationship existed between identification with colleagues and threat, although a positive relationship was found between social support and threat. Interestingly, without an established direction of causation, this could suggest that those who are more threatened seek more support. Caution should be applied when interpretating the strength of these findings given the relatively small relationships found. Although, while larger samples increase statistical power, they tend to lead to weaker correlation coefficients which may explain these current findings [91]. No other significant relationships were found in accordance with the hypotheses.

Evidence was also found for H3, in that females with greater identification with colleagues and lower threat was related to less perceived stress. These findings coincide with Slater et al. [42] postulations and the sociopsychobio model [46], which suggests social identity processes are important to help buffer against stress by altering appraisal processes and increasing the likelihood and effectiveness of social support. Specifically, it was proposed that social identification can influence the primary appraisal by providing a common interpretive framework [92]. In other words, members of a group who share common perspectives on the situation are more likely to interpret it in similar ways. For instance, those group members who have a shared identity when faced with a stressful situation change from the individual to group level, (e.g., “could this be dangerous to me?” to “could this be dangerous to us?”) [55]. In this sense, like the proverbial saying ‘a problem shared is a problem halved’, it may be possible that moving from an individual to a more group level will result in a lowering of a perceived demands and threat appraisal. Interestingly, only identification with colleagues, rather than identification with the organisation came out as a significant predictor of stress. This could be because in response to a stressful situation those members closest to the individual (i.e., colleagues) are considered more influential in helping to cope with the stressor than at organisation level. This is perhaps more pertinent in those larger organisations where the group memberships are not as salient as groups among colleagues. Past researchers have found that individuals tend to report greater levels of identification within teams and role relationships than with an organisation as a whole [93, 94]. Future researchers could explore the differing levels of group identification in the workplace and the effects it has on stress and challenge and threat responses.

Contrary to our hypothesis, neither social support nor challenge were significant predictors of perceived stress in the current data. The bivariate analysis also revealed no significant relationships. It would appear that this observation goes against the buffering effect of social support on stress [28]. Notwithstanding, these findings highlight the variability in individual’s appraisal of stressful events and that certain types of social support may not be useful in reducing perceived stress. Given that challenge and threat states are the resulting appraisal of the stressful event, these states do not advocate an increase or reduction in the perceptions of stress, which may explain why challenge did not predict perceived stress. To illustrate, an individual can still perceive high levels of stress, yet still feel they have appropriate resources to outweigh the demands and elicit a challenge state. These findings may also be explained by possibility of response bias, whereby participants tend to give more favourable answers to the items. For example, compared with females, males are more likely to report lower levels of social support due to their male role expectations [95]. As such, caution should be applied when interpreting these findings given the drawbacks of self-report measures.

In support of H4, we found that males and having greater levels of social identification, social support, and lower threat, was associated with greater life satisfaction. These findings are consistent with previous literature which have suggested that social identification and social support can have positive effects to wider health and wellbeing outcomes including life satisfaction [24, 96, 97]. It is considered that group identification can help buffer an individual from everyday stressors by creating a sense of meaning and increasing the likelihood of social support and in turn enhancing satisfaction with life [98].

The finding that greater perceived stress and lower social identification and life satisfaction were related to greater turnover intentions, also supported the hypothesis (H5). Researchers have supported the causal link between perceived stress and turnover intentions, identifying burnout as an important moderator among soccer officials [99], paediatricians [100] and student midwives [101]. Turnover intentions could be explained by the employee’s need to escape from unsatisfactory work conditions (i.e., job stress or feeling unsupported) [58], and meta-analytic evidence has revealed a strong correlation between turnover intentions and actual turnover [102]. Individuals with high levels of identification to their organization are likely to work harder towards achieving organizational goals, be more loyal and committed, and are therefore more likely to remain within their organization, when compared with those with lower identification [48, 58, 93, 103]. In other words, high identification at work is likely to reduce turnover intentions because the group is an important part of one’s self-concept, providing meaning and purpose and creating a sense of togetherness. Therefore, high identification could help buffer against some of the job demands and those environments which foster greater levels of identification with an organization, as well as among employees, should be encouraged. It is worth noting that the current data came from a sample which included a variety of service, private and education sector workers, which helps to generalize the findings across different industries. Taken together, given that high turnover can lead to significant economic, organizational, and service delivery consequences [104], these findings offer important implications for improving stress management techniques, increasing employee identification, and thus reducing turnover intentions.

There were no significant predictors of absenteeism (H6), although support was found for H7, in that females, along with having higher identification with the organisation and greater life satisfaction, with lower perceived stress were related to greater job performance. This finding could be explained in the literature as identification is seen to motivate group members to work for the groups interests, which in turn is seen to influence performance outcomes [47]. Instead of solely motivated to perform for themselves, there is a shift towards group-oriented effort and applying themselves on behalf of the group. For example, in a series of experiments [105] found that when group membership is salient, participants performed better on both brainstorming and simple motor tasks than those in the low salient conditions. It is thought that increasing the salience of an individual’s group membership will reduce the effects of social loafing and increase motivation and increased performance outcomes. Although it is worthwhile noting that performance in the current study was self-rated, so more holistic measures of performance could be examined in future research.

Despite the current findings, the present research is not without limitations which offers ideas for future researchers. First, establishing causation or directionality with cross-sectional studies can be difficult. It could be for example, that those with a greater identification are more likely to engage in more challenging/stressful situations, or those with greater life satisfaction will have the perception of higher identification and perceived social support. Researchers could examine these relationships with longitudinal research designs which would enable exploration into the moderating role of the social factors between challenge and threat and perceived stress and life satisfaction. Second, caution should be applied when interpreting the results given the self-report nature of the measures due to drawbacks such as response bias [106]. In line with this, participants were asked to recall their most stressful event over the last three months by completing the ALE-scale. Although, it is unknown the true intensity of the event or the accuracy of memory recall given that it can be impaired following stressful events [107]. Further, cognitive appraisal of challenge and threat can occur both consciously and unconsciously [12] and so capturing these through self-report raises concerns. Researchers should continue to adopt the objective cardiovascular framework of challenge and threat in more experimental designs to explore how social factors can influence challenge and threat states. It should also be noted the relatively low internal consistency scores for the ALE-scale, perceived stress, and the turnover intentions measure. While all considered acceptable as greater than 0.6 [108], this could be a result of the heterogeneity of the sample. Third, the current study did not measure the resource appraisals within the TCTSA [13] nor the postulations within revised 2 X 2 bifurcation theory of challenge and threat (TCTSA-R [20]). Therefore, without measures of Lazarus’ primary appraisals (i.e., motivational relevance & goal congruence), we cannot examine the TCTSA-R in the current research which would allow for a greater understanding of the influence of the social factors on the stress response.

Despite these limitations, we feel the current study contributes to the literature in several ways. First, from a theoretical perspective, we empirically examined how social factors (social support and social identification) can be related to challenge and threat, which addresses calls within recent theory (i.e., TCTSA-R). In this sense, our contribution supports the inclusion of the social factors in contemporary stress theory which also aligns with the framework proposed in the sociopsychobio model of health [46]. Second, we collected data across a range of different occupations to represent both private and public sector workers, which addresses calls to gather data beyond a single organization (e.g., [58, 109]). Third, and from a practical perspective, our study suggests that organizations should aim to foster a sense of identification given its positive associations with social support, life satisfaction, job performance, and the negative associations with perceptions of stress (and threat) and turnover intentions.

To conclude, the present study provides some evidence to demonstrate the role that social support and social identification can have on perceived stress and related outcomes (i.e., life satisfaction, turnover intentions, and job performance). There was also some initial evidence to draw a connection to challenge and threat states which has been scant in the literature. As Haslam [43] put it “Groups are thus a source of stress, but they can also be the key to overcoming it” (p. 191). In other words, the groups that we belong to can play an important role in how stress is appraised. To support the results from the current research, further studies need to be carried out using different population samples across other domains (i.e., sport and exercise, academia, leisure groups) to further understand the role that social factors play in the human stress response.

## Supporting information

S1 Table Participants job title. (DOCX)

S1 File Dataset. (SAV)

## References

[1] Leka S, Griffiths A, Cox T. Work organisation and Stress: Protecting Workers Health. World Health Organization, Institute of Work, Health & Organizations. 2003.

[2] Semmer NK. Individual Differences, Work Stress and Health. In: The Handbook of Work and Health Psychology: Second Edition. 2004.

[3] Johnstone M, Feeney JA. Individual differences in responses to workplace stress: the contribution of attachment theory. J Appl Soc Psychol. 2015 Jul;45(7):412–24.

[4] Halbesleben JRB, Buckley MR. Burnout in organizational life. J Manage. 2004.

[5] Health and Safety Executive. Work-related stress, anxiety or depression statistics in Great Britain, 2022 [Internet]. 2022 [cited 2023 Mar 24]. https://www.hse.gov.uk/statistics/

[6] Health and Safety Executive. Supplementary analysis of Costs to Britain data: using existing ill health appraisal values to estimate illustrative costs of work-related musculoskeletal disorders and stress. 2016;3–8.

[7] Bakker AB, Demerouti E. The Job Demands-Resources model: State of the art. Vol. 22, Journal of Managerial Psychology. Emerald Group Publishing Limited; 2007. p. 309–28.

[8] Lepine JA, Podsakoff NP, Lepine MA. A Meta-Analytic Test of the Challenge Stressor–Hindrance Stressor Framework: An Explanation for Inconsistent Relationships Among Stressors and Performance. Academy of Management Journal. 2005 Oct;48(5):764–75.

[9] Pearsall MJ, Ellis APJ, Stein JH. Coping with challenge and hindrance stressors in teams: Behavioral, cognitive, and affective outcomes. Organ Behav Hum Decis Process. 2009 May 1;109(1):18–28.

[10] Lazarus RS, Folkman S. Stress, Appraisal and coping. New York: Springer; 1984.

[11] Lazarus RS, Launier R. Stress-Related Transactions between Person and Environment. In: Perspectives in Interactional Psychology. 1978. p. 287–327.

[12] Blascovich J, Mendes WB. Challenge and threat appraisals: The role of affective cues. Feeling and thinking: The role of affect in social cognition. Studies in emotion and social interaction, second series. 2000. p. 59–82.

[13] Jones M, Meijen C, McCarthy PJ, Sheffield D. A theory of challenge and threat states in athletes. Int Rev Sport Exerc Psychol [Internet]. 2009;2(2):161–80. Available from: 10.1080/17509840902829331

[14] Mendes WB, Major B, McCoy S, Blascovich J. How Attributional Ambiguity Shapes Physiological and Emotional Responses to Social Rejection and Acceptance. J Pers Soc Psychol. 2008;94(2):278–91. doi: 10.1037/0022-3514.94.2.278 18211177 PMC2535927

[15] Zanstra YJ, Johnston DW, Rasbash J. Appraisal predicts hemodynamic reactivity in a naturalistic stressor. International Journal of Psychophysiology. 2010;77(1):35–42. doi: 10.1016/j.ijpsycho.2010.04.004 20417669 PMC3131091

[16] Seery MD, Weisbuch M, Hetenyi MA, Blascovich J. Cardiovascular measures independently predict performance in a university course. Psychophysiology. 2010;47(3):535–9. doi: 10.1111/j.1469-8986.2009.00945.x 20030765

[17] Vine SJ, Freeman P, Moore LJ, Chandra-Ramanan R, Wilson MR. Evaluating stress as a challenge is associated with superior attentional control and motor skill performance: Testing the predictions of the biopsychosocial model of challenge and threat. J Exp Psychol Appl. 2013;19(3):185–94. doi: 10.1037/a0034106 24059821

[18] Blascovich J, Mendes WB, Vanman E, Dickerson S. Social psychophysiology for social and personality psychology. Book. London: SAGE Publications Ltd.; 2011. 1–124 p.

[19] Seery MD. Challenge or threat? Cardiovascular indexes of resilience and vulnerability to potential stress in humans. Neurosci Biobehav Rev. 2011;35(7):1603–10. doi: 10.1016/j.neubiorev.2011.03.003 21396399

[20] Meijen C, Turner M, Jones M v., Sheffield D, McCarthy P. A Theory of Challenge and Threat States in Athletes: A Revised Conceptualization. Front Psychol. 2020 Feb 6;11:126. doi: 10.3389/fpsyg.2020.00126 32116930 PMC7016194

[21] Folkman S, Moskowitz JT. Coping: Pitfalls and Promise. Annu Rev Psychol. 2004 Feb 12;55(1):745–74. doi: 10.1146/annurev.psych.55.090902.141456 14744233

[22] Baumeister RF, Leary MR. The need to belong: desire for interpersonal attachments as a fundamental human motivation. Psychol Bull. 1995;117(3):497–529. 7777651

[23] Deci EL, Ryan RM. The “what” and “why” of goal pursuits: Human needs and the self-determination of behavior. Psychol Inq. 2000;11(4):227–68.

[24] Uchino BN. Understanding the Links Between Social Support and Physical Health: A Life-Span Perspective With Emphasis on the Separability of Perceived and Received Support. Perspectives on Psychological Science. 2009;4(3):236–55. doi: 10.1111/j.1745-6924.2009.01122.x 26158961

[25] Lin N, Ensel WM, Simeone RS, Kuo W. Social Support, Stressful Life Events, and Illness: A Model and an Empirical Test. J Health Soc Behav. 1979 Jun;20(2):108. 479524

[26] House J. Work Stress and Social Support. Reading: Addison-Wesley Pub. Co; 1981.

[27] Cutrona CE, Russell DW. Type of Social Support and Specific Stress: Toward a Theory of Optimal Matching. In: Social support: An Interactional View. 1990. p. 319–61.

[28] Cohen S, Wills TA. Stress, social support, and the buffering hypothesis. Psychol Bull [Internet]. 1985 Sep;98(2):310–57. Available from: http://doi.apa.org/getdoi.cfm?doi=10.1037/0033-2909.98.2.310 3901065

[29] Haber MG, Cohen JL, Lucas T, Baltes BB. The relationship between self-reported received and perceived social support: A meta-analytic review. Am J Community Psychol. 2007;39(1–2):133–44. doi: 10.1007/s10464-007-9100-9 17308966

[30] Helgeson VS. Two Important Distinctions in Social Support: Kind of Support and Perceived Versus Received1. J Appl Soc Psychol. 1993 May 1;23(10):825–45.

[31] Beals KP, Peplau LA, Gable SL. Stigma management and well-being: the role of perceived social support, emotional processing, and suppression. Pers Soc Psychol Bull. 2009;35:867–79. doi: 10.1177/0146167209334783 19403792

[32] Cohen S, McKay G. Social support, stress, and the buffering hypothesis: A theoretical analysis. Vol. 4, Handbook of psychology and health. 1984. p. 253–67.

[33] Berkman LF, Leo-Summers L, Horwitz RI. Emotional support and survival after myocardial infarction: A prospective, population-based study of the elderly. Ann Intern Med. 1992;117(12):1003–9. 1443968 10.7326/0003-4819-117-12-1003

[34] Ell K, Nishimoto R, Mediansky L, Mantell J, Hamovitch M. Social relations, social support and survival among patients with cancer. J Psychosom Res. 1992;36(6):531–41. doi: 10.1016/0022-3999(92)90038-4 1640391

[35] Lee M, Rotheram-Borus MJ. Challenges associated with increased survival among parents living with HIV. Am J Public Health. 2001;91(8):1303–9. doi: 10.2105/ajph.91.8.1303 11499123 PMC1446765

[36] Kong F, You X. Loneliness and Self-Esteem as Mediators Between Social Support and Life Satisfaction in Late Adolescence. Soc Indic Res. 2013.

[37] Holt-Lunstad J, Smith TB, Layton JB. Social relationships and mortality risk: A meta-analytic review. Vol. 7, PLoS Medicine. 2010. 10.1371/journal.pmed.1000316 PMC2910600 20668659

[38] Uchino BN. Social support and physical health: Understanding the health consequences of relationships. Social Support and Physical Health: Understanding the Health Consequences of Relationships. 2004. 1–222 p.

[39] Patterson GT. Examining the effects of coping and social support on work and life stress among police officers. J Crim Justice. 2003 May;31(3):215–26.

[40] Skaalvik EM, Skaalvik S. Dimensions of Teacher Self-Efficacy and Relations With Strain Factors, Perceived Collective Teacher Efficacy, and Teacher Burnout. J Educ Psychol. 2007;99(3):611–25.

[41] Dixon M, Turner MJ, Gillman J. Examining the relationships between challenge and threat cognitive appraisals and coaching behaviours in football coaches. J Sports Sci. 2016 Dec 26;1–7. doi: 10.1080/02640414.2016.1273538 28019726

[42] Slater MJ, Evans AL, Turner MJ. Implementing a Social Identity Approach for Effective Change Management. Journal of Change Management. 2016 Jan 2;16(1):18–37.

[43] Haslam AS. Psychology in Organizations:The Social Identity Approach [Internet]. 2nd ed. London: SAGE Publications Ltd.; 2004. 17–39 p. https://sk.sagepub.com/books/psychology-in-organizations

[44] Tajfel H, Turner JC. An integrative theory of intergroup conflict. In: S Worchel, & WG Austin: The social psychology of intergroup relations. Chicago: Nelson.; 1979. p. 33–47.

[45] Turner JC, Hogg MA, Oakes PJ, Reicher SD, Wetherell MS. Rediscovering the Social Group: A Self-Categorization Theory. Oxford: Blackwell; 1987. 42–67 p.

[46] Haslam SA, Haslam C, Jetten J, Cruwys T, Bentley S. Group life shapes the psychology and biology of health: The case for a sociopsychobio model. Soc Personal Psychol Compass [Internet]. 2019 Aug 30;13(8):1–16. Available from: https://onlinelibrary.wiley.com/doi/abs/10.1111/spc3.12490

[47] Van Knippenberg D. Work motivation and performance: A social identity perspective. Applied Psychology. 2000.

[48] Riketta M, Dick R Van. Foci of attachment in organizations: A meta-analytic comparison of the strength and correlates of workgroup versus organizational identification and commitment. J Vocat Behav. 2005.

[49] Cruwys T, Haslam SA, Dingle GA, Haslam C, Jetten J. Depression and Social Identity: An Integrative Review. Personality and Social Psychology Review [Internet]. 2014;18(3):215–38. Available from: http://psr.sagepub.com/cgi/doi/10.1177/1088868314523839 24727974 10.1177/1088868314523839

[50] Ng TWH, Feldman DC. Long work hours: a social identity perspective on meta-analysis data. J Organ Behav. 2008 Oct;29(7):853–80.

[51] Steffens NK, Haslam SA, Schuh SC, Jetten J, van Dick R. A Meta-Analytic Review of Social Identification and Health in Organizational Contexts. Personality and Social Psychology Review. 2017. doi: 10.1177/1088868316656701 27388779

[52] Haslam C, Jetten J, Cruwys T, Dingle GA, Haslam SA. The New Psychology of Health. The New Psychology of Health. 2018.

[53] Haslam SA, O’Brien A, Jetten J, Vormedal K, Penna S. Taking the strain: Social identity, social support, and the experience of stress. Br J Soc Psychol [Internet]. 2005;44(Pt 3):355–70. Available from: http://www.ncbi.nlm.nih.gov/pubmed/16238844 16238844 10.1348/014466605X37468

[54] Bruner MW, McLaren C, Swann C, Schweickle MJ, Miller A, Benson A, et al. Exploring the Relations between Social Support and Social Identity in Adolescent Male Athletes. Res Q Exerc Sport. 2020 Jun 3;00(00):1–7. doi: 10.1080/02701367.2020.1737629 32491971

[55] Ketturat C, Frisch JU, Ullrich J, Häusser JA, van Dick R, Mojzisch A. Disaggregating Within- and Between-Person Effects of Social Identification on Subjective and Endocrinological Stress Reactions in a Real-Life Stress Situation. Pers Soc Psychol Bull [Internet]. 2016 Feb 18;42(2):147–60. Available from: http://www.ncbi.nlm.nih.gov/pubmed/26586666 26586666 10.1177/0146167215616804

[56] Haslam SA, Reicher SD, Levine M. When other people are heaven, when other people are hell: How social identity determines the nature and impact of social support. In: The Social Cure: Identity, Health and Well-Being. 2012.

[57] Frisch JU, Hausser JA, van Dick R, Mojzisch A. The Social Dimension of Stress: Experimental Manipulations of Social Support and Social Identity in the Trier Social Stress Test. J Vis Exp. 2015;(105). doi: 10.3791/53101 26649856 PMC4692737

[58] Avanzi L, Fraccaroli F, Sarchielli G, Ullrich J, van Dick R. Staying or leaving:A combined social identity and social exchange approach to predicting employee turnover intentions. International Journal of Productivity and Performance Management. 2014 Apr 8;63(3):272–89.

[59] Allen KM, Blascovich J, Tomaka J, Kelsey RM. Presence of human friends and pet dogs as moderators of autonomic responses to stress in women. J Pers Soc Psychol. 1991;61(4):582–9. doi: 10.1037//0022-3514.61.4.582 1960650

[60] Anthony JL, O’Brien WH. An evaluation of the impact of social support manipulations on cardiovascular reactivity to laboratory stressors. Behavioral Medicine. 1999 Jan;25(2):78–87. doi: 10.1080/08964289909595740 10401537

[61] Bolger N, Amarel D. Effects of social support visibility on adjustment to stress: Experimental evidence. J Pers Soc Psychol. 2007;92(3):458–75. doi: 10.1037/0022-3514.92.3.458 17352603

[62] Maisel NC, Gable SL. The paradox of received social support: The importance of responsiveness. Psychol Sci. 2009;20(8):928–32. doi: 10.1111/j.1467-9280.2009.02388.x 19549083

[63] Shrout PE, Herman CM, Bolger N. The costs and benefits of practical and emotional support on adjustment: A daily diary study of couples experiencing acute stress. Pers Relatsh. 2006;13(1):115–34.

[64] Kaufmann GM, Beehr TA. Interactions Between Job Stressors and Social Support. Some Counterintuitive Results. Journal of Applied Psychology. 1986. 3745079

[65] Fenlason KJ, Beehr TA. Social support and occupational stress: Effects of talking to others. J Organ Behav. 1994.

[66] Slater MJ, Turner MJ, Evans AL, Jones MV. Capturing hearts and minds: The influence of relational identification with the leader on followers’ mobilization and cardiovascular reactivity. Leadersh Q. 2018 Jun;29(3):379–88.

[67] Dixon M, Turner MJ. Stress appraisals of UK soccer academy coaches: an interpretative phenomenological analysis. Qual Res Sport Exerc Health. 2018.

[68] Miller AJ, Slater MJ, Turner MJ. Coach identity leadership behaviours are positively associated with athlete resource appraisals: The mediating roles of relational and group identification. Psychol Sport Exerc. 2020.

[69] Blascovich J. Challenge and threat appraisal. In: Handbook of approach and avoidance motivation. New York: Psychology Press; 2008. p. 432–44.

[70] Moore LJ, Vine SJ, Wilson MR, Freeman P. Examining the antecedents of challenge and threat states: The influence of perceived required effort and support availability. International journal of psychophysiology [Internet]. 2014 Aug [cited 2020 Apr 30];93(2):267–73. Available from: http://www.ncbi.nlm.nih.gov/pubmed/24867434 24867434 10.1016/j.ijpsycho.2014.05.009

[71] Elangovan AR. Causal ordering of stress, satisfaction and commitment, and intention to quit: A structural equations analysis. Leadership & Organization Development Journal. 2001.

[72] Webster JR, Beehr TA, Love K. Extending the challenge-hindrance model of occupational stress: The role of appraisal. J Vocat Behav. 2011 Oct;79(2):505–16.

[73] Brunner B, Igic I, Keller AC, Wieser S. Who gains the most from improving working conditions? Health-related absenteeism and presenteeism due to stress at work. European Journal of Health Economics. 2019. doi: 10.1007/s10198-019-01084-9 31309366 PMC6803571

[74] Palan S, Schitter C. Prolific.ac—A subject pool for online experiments. J Behav Exp Finance. 2018.

[75] Ferguson E, Matthews G, Cox T. The Appraisal of Life Events (ALE) scale: Reliability and validity. Br J Health Psychol. 1999.

[76] Postmes T, Haslam SA, Jans L. A single-item measure of social identification: Reliability, validity, and utility. British Journal of Social Psychology. 2013;52(4):597–617. doi: 10.1111/bjso.12006 23121468

[77] Zimet GD, Dahlem NW, Zimet SG, Gordon K, Farley GK. The Multidimensional Scale of Perceived Social Support The Multidimensional Scale of Perceived Social Support. J Pers Assess. 1998;52(1):37–41. 10.1080/00223891.1990.9674095 2280326

[78] Osman A, Lamis DA, Freedenthal S, Gutierrez PM, McNaughton-Cassill M. The Multidimensional Scale of Perceived Social Support: Analyses of Internal Reliability, Measurement Invariance, and Correlates Across Gender. J Pers Assess. 2014 Jan 2;96(1):103–12. doi: 10.1080/00223891.2013.838170 24090236

[79] Büssing A, Fischer J, Haller A, Heusser P, Ostermann T, Matthiessen P. Validation of the brief multidimensional life satisfaction scale in patients with chronic diseases. Eur J Med Res. 2009;14(4):171. doi: 10.1186/2047-783x-14-4-171 19380290 PMC3401007

[80] Seligson JL, Huebner ES, Valois RF. Preliminary validation of the Brief Multidimensional Students’ Life Satisfaction Scale (BMSLSS). Soc Indic Res. 2003.

[81] Abubakar A, van de Vijver F, Alonso-Arbiol I, He J, Adams B, Aldhafri S, et al. Measurement Invariance of the Brief Multidimensional Student’s Life Satisfaction Scale Among Adolescents and Emerging Adults Across 23 Cultural Contexts. J Psychoeduc Assess. 2016 Feb 30;34(1):28–38.

[82] Cohen S, Kamarck TW, Mermelstein R. A global measure of perceived stress. J Health Soc Behav. 1983;24(4):385–96. 6668417

[83] Lee EH. Review of the Psychometric Evidence of the Perceived Stress Scale. Asian Nurs Res (Korean Soc Nurs Sci). 2012 Dec 1;6(4):121–7. doi: 10.1016/j.anr.2012.08.004 25031113

[84] Colarelli SM. Methods of communication and mediating processes in realistic job previews. Journal of Applied Psychology. 1984.

[85] Kessler RC, Barber C, Beck A, Berglund P, Cleary PD, McKenas D, et al. The World Health Organization Health and Work Performance Questionnaire (HPQ). J Occup Environ Med. 2003. doi: 10.1097/01.jom.0000052967.43131.51 12625231

[86] Dyrbye LN, Shanafelt TD, Johnson PO, Johnson LA, Satele D, West CP. A cross-sectional study exploring the relationship between burnout, absenteeism, and job performance among American nurses. BMC Nurs. 2019 Dec 21;18(1):57. doi: 10.1186/s12912-019-0382-7 31768129 PMC6873742

[87] Graham JW. Missing Data Analysis: Making It Work in the Real World. Annu Rev Psychol. 2009. doi: 10.1146/annurev.psych.58.110405.085530 18652544

[88] Salkind CNJ, Effect H, Horner RH, Spaulding SA, Effect H, Salkind CNJ. Encyclopedia of Research Design Encyclopedia of research design. Regression Coefficient. Encyclopedia of Research Design. 2010. 1499–1503 p.

[89] Smith M. Research methods in accounting. Third edition. Sage Publications Ltd; 2014. 278 p.

[90] Avanzi L, Schuh SC, Fraccaroli F, van Dick R. Why does organizational identification relate to reduced employee burnout? The mediating influence of social support and collective efficacy. Work Stress [Internet]. 2015 Jan 2;29(1):1–10. Available from: http://ezproxy.shu.edu/login?url=http://search.ebscohost.com/login.aspx?direct=true&db=s3h&AN=101347942&site=eds-live

[91] Armstrong RA. Is there a large sample size problem? Ophthalmic and Physiological Optics. 2019. 10.1111/opo.12618 30994198

[92] Haslam SA, Reicher SD. Stressing the group: Social identity and the unfolding dynamics of responses to stress. Journal of Applied Psychology [Internet]. 2006;91(5):1037–52. Available from: http://doi.apa.org/getdoi.cfm?doi=10.1037/0021-9010.91.5.1037 16953766 10.1037/0021-9010.91.5.1037

[93] Riketta M, Nienaber S. Multiple identities and work motivation: The role of perceived compatibility between nested organizational units. British Journal of Management. 2007.

[94] Sluss DM, Ployhart RE, Cobb MG, Ashforth BE. Generalizing newcomers’ relational and organizational identifications: Processes and prototypicality. Academy of Management Journal. 2012.

[95] Wester SR, Christianson HF, Vogel DL, Wei M. Gender Role Conflict and Psychological Distress: The Role of Social Support. Psychol Men Masc. 2007.

[96] Cohen S, Gottlieb B, Underwood L. Social relationships and health. In: Measuring and intervening in social support. 2000. p. 3–25.

[97] Sani F, Herrera M, Wakefield JRH, Boroch O, Gulyas C. Comparing social contact and group identification as predictors of mental health. British Journal of Social Psychology. 2012. doi: 10.1111/j.2044-8309.2012.02101.x 22550954

[98] Jetten J, Haslam SA, Iyer A, Haslam C. Turning to Others in Times of Change. In: The Psychology of Prosocial Behavior. 2009.

[99] Taylor AH, Daniel J v., Leith L, Burke RJ. Perceived stress, psychological burnout and paths to turnover intentions among sport officials. J Appl Sport Psychol. 1990.

[100] Grossman Z, Chodick G, Kushnir T, Cohen HA, Chapnick G, Ashkenazi S. Burnout and intentions to quit the practice among community pediatricians: Associations with specific professional activities. Isr J Health Policy Res. 2019. doi: 10.1186/s13584-018-0268-2 30609943 PMC6318951

[101] Eaves JL, Payne N. Resilience, stress and burnout in student midwives. Nurse Educ Today. 2019. 10.1016/j.nedt.2019.05.012 31153089

[102] Rubenstein AL, Eberly MB, Lee TW, Mitchell TR. Surveying the forest: A meta-analysis, moderator investigation, and future-oriented discussion of the antecedents of voluntary employee turnover. Pers Psychol. 2018 Mar;71(1):23–65.

[103] Haslam SA, Van Dick R. A social identity approach to workplace stress. In: Social Psychology and Organizations. 2011.

[104] Allisey AF, Noblet AJ, Lamontagne AD, Houdmont J. Testing a Model of Officer Intentions to Quit: The Mediating Effects of Job Stress and Job Satisfaction. Crim Justice Behav. 2014.

[105] van Dick R, Stellmacher J, Wagner U, Lemmer G, Tissington PA. Group membership salience and task performance. Journal of Managerial Psychology. 2009.

[106] Rosenman R, Tennekoon V, Hill LG. Measuring bias in self-reported data. Int J Behav Healthc Res. 2011;2(4):320. doi: 10.1504/IJBHR.2011.043414 25383095 PMC4224297

[107] Kuhlmann S, Wolf OT. Arousal and cortisol interact in modulating memory consolidation in healthy young men. Behavioral Neuroscience. 2006. doi: 10.1037/0735-7044.120.1.217 16492134

[108] Cronbach LJ. Coefficient alpha and the internal structure of tests. Psychometrika. 1951 Sep;16(3):297–334.

[109] Avanzi L, Perinelli E, Mariani MG. The effect of individual, group, and shared organizational identification on job satisfaction and collective actual turnover. Eur J Soc Psychol. 2023 Apr 5.

