# Supplementary material for: The role of social support and social identification on challenge and threat cognitive appraisals, perceived stress, and life satisfaction in workplace employees
Source: PLoS One. 2023 Jul 12;18(7):e0288563. doi: 10.1371/journal.pone.0288563 (PMC10337949; doi:10.1371/journal.pone.0288563)
Supplement: S1 Table — (DOCX) [file pone.0288563.s001.docx]

**S1 Table: Participants Job Title**

| **Job Title** | **N** | **Job Category** |
| --- | --- | --- |
| Mental health nurse | 1 | 1 |
| Nurse | 3 | 1 |
| Nurse manager | 1 | 1 |
| Social worker | 1 | 1 |
| Police Staff | 1 | 1 |
| Local Government Officer | 1 | 1 |
| Senior care assistant | 1 | 1 |
| Community support officer | 1 | 1 |
| Speech and Language Therapist (SLT) (NHS) | 69 | 1 |
| Speech and language Therapy Manager/Clinician (NHS) | 6 | 1 |
| SLT Support Worker/Assistant (NHS) | 8 | 1 |
| Secretary/Admin Coordinator (NHS) | 1 | 1 |
| Allied Health Professional (AHP) (NHS) | 1 | 1 |
| AHP support worker (NHS) | 1 | 1 |
| Technical Instructor (NHS) | 1 | 1 |
| Service lead (NHS) | 2 | 1 |
| Community fire safety officer | 1 | 1 |
| Customer contact advisor (Fire & Rescue) | 1 | 1 |
| Director of Response (Fire & Rescue) | 1 | 1 |
| Support Staff (Fire & Rescue) | 4 | 1 |
| Finance (Fire & Rescue) | 1 | 1 |
| Prevent Team Leader (Fire & Rescue) | 2 | 1 |
| Deputy chief (Fire & Rescue) | 1 | 1 |
| Director (Fire & Rescue) | 1 | 1 |
| Media Officer (Fire & Rescue) | 1 | 1 |
| Marketing (Fire & Rescue) | 1 | 1 |
| Trainer (Fire & Rescue) | 1 | 1 |
| Protect Coordinator (Fire & Rescue) | 1 | 1 |
| Watch Manager (Fire & Rescue) | 1 | 1 |
| Risk Manager (Fire & Rescue) | 1 | 1 |
| HR Apprentice/ HR Administration Assistant (Fire & Rescue) | 1 | 1 |
| Human Resources (Fire & Rescue) | 2 | 1 |
| Water Manager (Fire & Rescue) | 1 | 1 |
| Instructor (Fire & Rescue) | 1 | 1 |
| Manager (unclassified) (Fire & Rescue) | 2 | 1 |
| Firefighter (Fire & Rescue) | 2 | 1 |
| Chief Fire Officer (Fire & Rescue) | 1 | 1 |
| Training Officer (RAF) | 1 | 1 |
| ATCIC Trainee (RAF) | 6 | 1 |
| JATCC Trainee (RAF) | 12 | 1 |
| Air Traffic Controller (RAF) | 2 | 1 |
| SAOC Trainee (RAF) | 1 | 1 |
| FOTF Trainee (RAF) | 3 | 1 |
| FOA (RAF) | 6 | 1 |
| FOAC trainee (RAF) | 2 | 1 |
| Flight operations (RAF) | 1 | 1 |
| Flight Operation Assistant (RAF) | 2 | 1 |
| Air Traffic Trainer (RAF) | 1 | 1 |
| ATCO (RAF) | 1 | 1 |
| Warrant Officer Class One (Royal Marines) | 1 | 1 |
| Tp SGT (Royal Marines) | 1 | 1 |
| Operations (Royal Marines) | 1 | 1 |
| Consultant anaesthetist | 2 | 1 |
| SAS Surgeon | 1 | 1 |
| Hospital doctor | 2 | 1 |
| Specialist registrar | 1 | 1 |
| GP | 1 | 1 |
| Consultant (Surgeon) | 1 | 1 |
| Paediatrician | 1 | 1 |
| Software developer | 1 | 2 |
| Software engineer | 1 | 2 |
| Director (unclassified) | 5 | 2 |
| Accounts assistant | 1 | 2 |
| Checkout operator | 3 | 2 |
| Claims handler | 1 | 2 |
| Officer | 1 | 2 |
| Business owner | 1 | 2 |
| Operations assistant | 1 | 2 |
| Project manager | 1 | 2 |
| Customer service manager | 2 | 2 |
| Personal Assistant | 1 | 2 |
| Manager (unclassified) | 14 | 2 |
| Assistant manager (unclassified) | 2 | 2 |
| Team leader (unclassified) | 7 | 2 |
| Call handler | 1 | 2 |
| Customer specialist | 2 | 2 |
| Storeman | 1 | 2 |
| Analyst (unclassified) | 4 | 2 |
| Paraplanner | 1 | 2 |
| Training (unclassified) | 1 | 2 |
| Accounts assistant | 2 | 2 |
| Freelance | 1 | 2 |
| Chef | 3 | 2 |
| Development consultant | 1 | 2 |
| Sales | 2 | 2 |
| Electrician | 1 | 2 |
| Administrator | 15 | 2 |
| Operations manager | 2 | 2 |
| Demand planner | 1 | 2 |
| Mortgage consultant | 1 | 2 |
| Warehouse operative | 1 | 2 |
| Road engineer | 1 | 2 |
| Money Advisor | 1 | 2 |
| Assistant practice manager | 1 | 2 |
| Probation | 1 | 2 |
| IT Manager | 1 | 2 |
| Research officer | 1 | 2 |
| Librarian | 2 | 2 |
| IT developer | 1 | 2 |
| Sales manager | 1 | 2 |
| Ambassador development coordinator | 1 | 2 |
| Veterinary nurse | 1 | 2 |
| Elearning developer | 1 | 2 |
| Managed service consultant | 1 | 2 |
| Transport planner | 1 | 2 |
| Highway engineer | 1 | 2 |
| Keyholder | 1 | 2 |
| Dog trainer | 1 | 2 |
| Operative | 1 | 2 |
| Logistics coordinator | 1 | 2 |
| Support & advice worker | 2 | 2 |
| Physiotherapist triage | 1 | 2 |
| EUC Analyst | 1 | 2 |
| Solicitor | 1 | 2 |
| Head of Marketing | 2 | 2 |
| Editor | 1 | 2 |
| Major donor fundraiser | 1 | 2 |
| Tour guide | 1 | 2 |
| Technical officer | 1 | 2 |
| HR | 2 | 2 |
| HR Manager | 2 | 2 |
| Marketing coordinator | 1 | 2 |
| Engineer | 1 | 2 |
| Senior policy advisor | 1 | 2 |
| Telecoms engineer | 1 | 2 |
| IT Consultant | 2 | 2 |
| Catering manager | 1 | 2 |
| Bar manager | 1 | 2 |
| Data analyst | 1 | 2 |
| Flavourist | 1 | 2 |
| Technician | 1 | 2 |
| Quality mail checker | 1 | 2 |
| Assistant (unclassified) | 1 | 2 |
| Plumber | 1 | 2 |
| Architectural assistant | 1 | 2 |
| Enquiries advisor | 1 | 2 |
| Forklift driver | 1 | 2 |
| Service advisor | 1 | 2 |
| Deeds and records assistant | 1 | 2 |
| Primary teacher | 4 | 3 |
| Trainee teacher | 7 | 3 |
| Associate teacher | 4 | 3 |
| Teacher (unclassified) | 22 | 3 |
| Dance teacher | 2 | 3 |
| English teacher | 4 | 3 |
| Geography teacher | 1 | 3 |
| PE teacher | 7 | 3 |
| Head of Performing Arts | 1 | 3 |
| Maths & PE teacher | 1 | 3 |
| PE & Business teacher | 1 | 3 |
| History teacher | 1 | 3 |
| Duty Officer in Education | 1 | 3 |
| Postgraduate researcher | 37 | 3 |
| Paramedic lecturer | 1 | 3 |
| Did not specify job role | 5 | 1 = 2; 2 = 2; 3 = 1) |

Note: Job categories: 1=Services (N=179), 2=Private (N=138), 3= Education (N=95),
